# Oral Administration of Egg- and Soy-Derived Lysophosphatidylcholine Mitigated Acetylcholine Depletion in the Brain of Scopolamine-Treated Rats

**DOI:** 10.3390/nu15163618

**Published:** 2023-08-17

**Authors:** Ryohei Tanaka-Kanegae, Hiroyuki Kimura, Koichiro Hamada

**Affiliations:** Saga Nutraceuticals Research Institute, Otsuka Pharmaceutical Co., Ltd. 5006-5 Aza Higashiyama, Yoshinogari-cho, Kanzaki-gun, Omagari, Saga 842-0195, Japan

**Keywords:** lysophosphatidylcholine, enzyme-modified lecithin, supplementation, brain, acetylcholine, choline, glycerophosphocholine, metabolism, scopolamine

## Abstract

Enzyme-modified lecithin that contains lysophosphatidylcholine (LPC) is generally recognized as safe. However, its potential as a functional ingredient has been less investigated than other choline (Ch)-containing compounds, such as glycerophosphocholine (GPC). Reports on the possibility of LPC functioning as a cholinergic precursor in vivo and on its kinetics are limited to docosahexaenoic acid-bound LPC. Herein, three experiments were performed to investigate these processes in scopolamine (SCO)-treated rats. First, an egg-derived LPC reagent was orally administered to rats, and brain acetylcholine (ACh), Ch, plasma Ch, and LPC were measured. Second, soy- and rapeseed-derived enzyme-modified lecithins and GPC were administered for comparison. Third, soy-derived enzyme-modified lecithins with different fat contents were administered for comparison. The LPC reagent mitigated SCO-induced ACh depletion at 500 mg/kg body weight and increased plasma Ch, but not LPC, concentrations. Additionally, soy-derived LPC-containing food additive counteracted brain ACh depletion similarly to GPC. Interestingly, plasma Ch and linoleoyl-LPC levels were higher when soy-derived LPC with a higher fat content was administered, whereas the plasma levels of palmitoyl-LPC decreased and those of total LPC remained constant. In conclusion, egg- and soy-derived LPC species function as cholinergic precursors in vivo, and future studies should explore this potential.

## 1. Introduction

**Figure 1:**
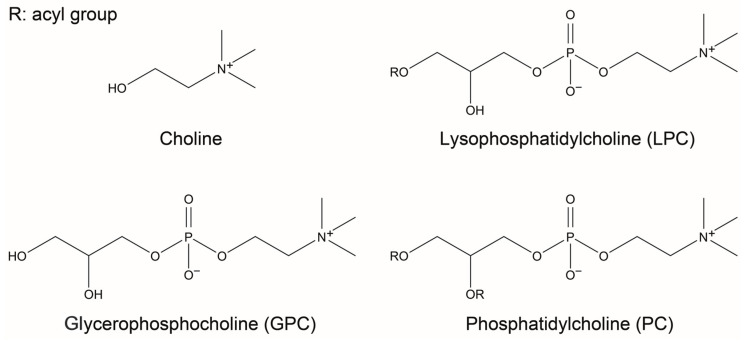
Chemical structures of LPC, GPC, and PC. LPC, lysophosphatidylcholine; GPC, glycerophosphocholine; PC, phosphatidylcholine.

Lysophosphatidylcholine (LPC), a phospholipid found in the organs of living organisms, such as blood and the brain, and in various foods, is composed of a glycerol backbone with a single acyl group at sn1 or sn2 and phosphorylcholine at sn3 [1,2]. Glycerophosphocholine (GPC) and phosphatidylcholine (PC) are choline (Ch)-containing compounds that are structurally similar to LPC (their chemical structures are shown in Figure 1), and their cholinergic activity has been rigorously investigated for their potential as a cognitive enhancer [3,4,5]. However, studies on LPC are lacking, with the exception of those on docosahexaenoic acid (DHA)-bound LPC [6,7,8], because LPC was reported to play an inflammatory role owing to its conversion to lysophosphatidic acid (LPA) under the action of autotaxin [9]. Nevertheless, the blood concentration of LPC is not necessarily associated with that of LPA [10,11], and LPC is essential for brain development (reviewed in [2]). Moreover, studies showed that LPC bound to a polyunsaturated fatty acid, such as DHA, had anti-inflammatory potential [8,12], and DHA-LPC supplementation increased brain ACh levels and improved cognitive performance in vivo [7,13]. Indeed, abnormal levels of saturated (palmitoyl and stearoyl) and monounsaturated (oleoyl) LPCs in the body are associated with inflammatory and pathogenic changes (reviewed in [11]); however, in vivo levels of these LPC species are firmly controlled under normal physiological conditions [14]. The safety of oral consumption of LPC is also reflected by the fact that enzyme-modified lecithin (also known as lysolecithin), which contains LPC, is generally recognized as safe (GRAS) [15]. Although LPC species, other than DHA-LPC, are the predominant forms among commercially available enzyme-modified lecithins [16], the effects of oral administration of these LPC species on brain function have been poorly investigated.

Scopolamine (SCO), a muscarinic acetylcholine (ACh) receptor antagonist that disrupts cholinergic neurotransmission, is frequently used as an in vivo experimental model to assess the cholinergic activity of test compounds and their effects on cognitive function. Ch-containing compounds [17], such as GPC and PC, have been evaluated using this model; however, LPC has not been investigated [18,19,20]. Furthermore, a relatively limited number of studies have been conducted on the in vivo kinetics of LPC compared with studies on that of other phospholipids [21]. Oral supplementation of LPC in the docosahexaenoic acid (DHA)-bound form increases blood DHA-LPC levels, and DHA-LPC in blood is transported across the blood-brain barrier by a DHA-LPC transporter [7,22]. However, the metabolic fates of other LPC species remain unclear.

This study aimed to assess the cholinergic activity of common LPC species from different sources by measuring the ACh content in the brain of SCO-treated rats. Moreover, LPC absorption and metabolism were examined by determining the plasma Ch and LPC levels. Three experiments were conducted. First, an egg-derived LPC reagent was orally administered, and the brain ACh and Ch and plasma Ch and LPC were determined. Second, soy- and rapeseed-derived enzyme-modified lecithins were administered and compared with GPC to evaluate their potential as functional ingredients. Third, soy-derived enzyme-modified lecithins with different fat contents were administered to investigate the effects of fat on LPC kinetics. Although the cholinergic hypothesis is quite old, an approach to the cholinergic system is still considered practical for the treatment of cognitive dysfunction [4]. The current study may provide new perspectives on LPC and enzyme-modified lecithins as candidates for functional foods and drugs.

## 2. Materials and Methods

### 2.1. Reagents

Egg-derived LPC reagent (obtained by hydrolysis with phospholipase A2, LPC purity > 97%; CAS RN^®^:9008-30-4) and GPC reagent (GPC purity > 99%; CAS RN^®^:28319-77-9) were obtained from Wako Pure Chemical Industries, Ltd. (Osaka, Japan), and SCO hydrobromide was purchased from Sigma-Aldrich (St. Louis, MO, USA). Isopropylhomocholine was provided by Eicom Corporation (Kyoto, Japan), and LPC standards (palmitoyl-, stearoyl-, oleoyl-, linoleoyl-, linolenoyl-, and nonadecanoyl-LPC) were obtained from Avanti Polar Lipids Inc. (Alabaster, AL, USA). LIPOID R LPC 20 (LPC purity: 26%) was provided by H. Holstein Co., Ltd. (Tokyo, Japan), and SLP-LPC70 (LPC purity: 59%) and SLP-PasteLyso (LPC purity: 9%) were provided by Tsuji Oil Mills Co., Ltd. (Mie, Japan). The fatty acid compositions of LPC in the reagent, LIPOID R LPC 20, SLP-LPC70, and SLP-PasteLyso (Table 1) were determined using a high-performance liquid chromatography (HPLC) system combined with an evaporative light-scattering detector. 

The information on the fat content of each enzyme-modified lecithin was provided by the manufacturers; LIPOID R LPC 20 contained less than 3% of triglyceride, while the SLP-LPC70 and SLP-PasteLyso used for the present study contained 5–6% and 55% acetone-soluble matter, respectively. Additionally, we confirmed that the PC contents of LIPOID R LPC 20, SLP-LPC70, and SLP-PasteLyso were 26%, 2%, and 2%, respectively.

### 2.2. Animals

Male 6-week-old Wistar rats were purchased from Charles River Laboratories Japan Inc. (Kanagawa, Japan) and housed separately in aluminum cages under specific pathogen-free conditions. The room was maintained at 23 ± 1 °C and 55 ± 10% relative humidity. The rats were allowed access to AIN-93G pellets (Oriental Yeast Co., Ltd., Tokyo, Japan) and sterilized water ad libitum with a 12-h light (7:00–19:00)/dark (19:00–7:00) cycle. The rats were acclimated to the environment for one week prior to the experiments. The number of animals used for each experiment was 10–11, 5–11, and 10–11 rats per group in Experiments 1, 2, and 3, respectively.

### 2.3. Protocol for Animal Experiments

The animal studies were reviewed and approved by the Animal Experiment Ethics Committee of Otsuka Pharmaceutical Co., Ltd. (Tokyo, Japan) and performed in accordance with the institutional guidelines.

#### 2.3.1. Experiment 1 (LPC Reagent)

Rats were fasted overnight. LPC reagent was dissolved in sterilized water and orally administered at 167, 500, and 1500 mg/kg body weight (BW; 20 mL solution/kg BW). SCO was intraperitoneally injected at 1 mg/kg BW 4.5 h after administration; after 1.5 h, the rat brain was fixated using a microwave applicator (TMW-6402C, Muromachi Kikai Co., Ltd., Tokyo, Japan) and the frontal cortex was separated. Blood was collected immediately after brain fixation and centrifuged (3000× *g*, 15 min, 4 °C) to obtain plasma for Ch and LPC measurements.

#### 2.3.2. Experiment 2 (LPC-Containing Food Additives and GPC)

The experiment was conducted using the same schedule as that in Experiment 1. Two LPC-containing food additives, LIPOID R LPC 20 and SLP-LPC70, which were distributed as enzyme-modified lecithin, were used in addition to GPC. The total Ch content (free plus [lyso]phospholipid forms) of the test food additives was quantified using LabAssay ™ Phospholipid (Wako Pure Chemical Industries, Ltd.). The test food additives and GPC were orally administered to the rats at 100 mg Ch equivalent/kg BW (2000, 1280, and 246 mg/kg BW for LIPOID R LPC 20, SLP-LPC70, and GPC, respectively). Following the same SCO treatment and fixation procedure as that in Experiment 1, the brains were harvested and divided into the frontal cortex, hippocampus, and striatum.

#### 2.3.3. Experiment 3 (LPC-Containing Food Additives with Different Fat Contents)

The experiment was conducted using the same schedule as that in the other experiments. Two soy-derived enzyme-modified lecithins, SLP-LPC70 and SLP-PasteLyso, were dissolved in pure water and administered at 500 mg LPC equivalent/kg BW (846 and 5556 mg/kg BW for SLP-LPC70 and SLP-PasteLyso, respectively). As SLP-LPC70 is the purified form of SLP-PasteLyso, its fatty acid composition is similar (Table 1), but its fat content is considerably different from SLP-PasteLyso (6% for SLP-LPC70 and 55% for SLP-PasteLyso). The frontal cortex, hippocampus, striatum, and blood were collected following the same SCO treatment and fixation procedure as in the other experiments. The blood was centrifuged (3000× *g*, 15 min, 4 °C) to obtain plasma for Ch and LPC measurements.

### 2.4. ACh and Ch Measurement

Sample pretreatment and measurement of ACh and Ch levels were performed as described previously [23]. The divided brain samples were sonicated in perchloric acid and centrifuged (15,000× *g*, 15 min, 4 °C). The supernatants were neutralized and mixed with isopropylhomocholine as the internal standard. The supernatants were delipidated twice with chloroform and filtered, and they were subsequently injected into the HPLC-electrochemical detection system. The determined values were then divided by the weight of the segmented brain. Plasma was pretreated similarly, and Ch concentration was determined.

### 2.5. LPC Measurement

After the addition of nonadecanoyl-LPC as an internal standard, plasma LPC was extracted using the Bligh and Dyer method [24]. The extract was dried under reduced pressure and reconstituted with methanol. After filtration using a 0.2-μm polytetrafluoroethylene membrane (Millex-LG; Millipore Corporation, Billerica, MA, USA), the filtrates were injected into the HPLC (Prominence; SHIMADZU CORPORATION, Kyoto, Japan) combined with an evaporative light scattering detector (ELSD-LTII, SHIMADZU CORPORATION). A Purospher^®^ STAR RP-18 endcapped (5 µm) LiChroCART^®^ (250 × 4 mm) was used as a separate column with a guard column (Merck KGaA, Darmstadt, Germany). The mobile phases were 75% acetonitrile with 0.1% formic acid (A) and 2-propanol (B), and the gradient conditions were: A:B = 100:0 for 15 min (1.0 mL/min), changed to 0:100 over 5 min, maintained at 0:100 for 15 min, then changed to 100:0 over 5 min (0.6 mL/min), and maintained at 100:0 for 10 min (1.0 mL/min). The settings for the detector were 40 °C, 350 kPa, and a gain of 12. The detected areas for different concentrations of palmitoyl-LPC standards were converted to a logarithmic scale to generate a fitting curve, and palmitoyl-, stearoyl-, oleoyl-, linoleoyl-, and linolenoyl-LPCs were quantified.

### 2.6. Statistical Analyses

Microwave irradiation was considered inadequate to fully deactivate enzymes related to ACh metabolism when the harvested brain appeared raw, and the calculated Ch concentration was 1.5 times higher than the average Ch concentration of the group. In these cases, data were excluded from the statistical analyses. The Ch value for exclusion was determined based on the SD of the values in the SCO-treated group. After standardization with a mean of 1, the 2SD was 0.34. Data are expressed as mean ± standard error of the mean (SEM). Analysis of covariance (ANCOVA) with rat BW as a covariate and Dunnett’s post hoc test were performed to assess significant differences between the groups. The SCO-alone condition was used as the standard. In addition, an unpaired *t*-test was used for Experiment 1 to assess the difference in plasma Ch levels between the two SCO-untreated groups as well as for Experiment 3. Moreover, the Jonckheere test (two-sided) was performed to assess the LPC dose dependency of plasma Ch levels. Differences with probability (*p*) values < 0.05 were considered statistically significant. SAS 9.4 (SAS Institute Inc., Cary, NC, USA) was used for statistical analyses.

## 3. Results

### 3.1. Experiment 1 (LPC Reagent)

**Figure 2:**
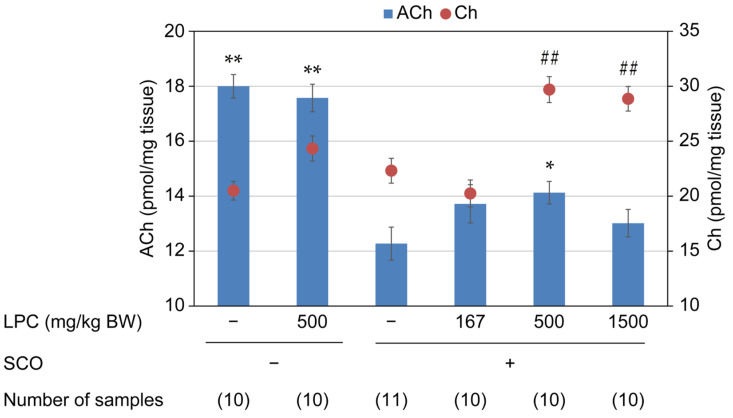
Orally administered LPC mitigated SCO-induced ACh depletion in the frontal cortex. ACh and Ch levels in the frontal cortex were quantified 6 h after LPC administration (167, 500, and 1500 mg/kg BW, p.o.)/1.5 h after SCO injection (1 mg/kg BW, i.p.). Data are presented as mean ± SEM. *: *p* < 0.05, **: *p* < 0.01 for ACh vs. LPC−, SCO+; ##: *p* < 0.01 for Ch vs. LPC−, SCO+. ACh, acetylcholine; BW, body weight; Ch, choline; SCO, scopolamine; SEM, standard error of the mean.

The ACh and Ch levels in the frontal cortex are shown in Figure 2. The SCO-alone administration group showed a significantly lower ACh level than the sham group (12.3 ± 0.6 pmol/mg tissue for LPC−, SCO+ vs. 18.0 ± 0.4 pmol/mg tissue for LPC−, SCO−; *p* < 0.0001), whereas the SCO plus 500 mg/kg LPC group showed a significantly higher ACh level than the SCO-alone group (14.1 ± 0.4 pmol/mg tissue; *p* = 0.044 vs. LPC−, SCO+). LPC at 500 mg/kg alone did not increase the ACh concentration. Similarly, the administration of SCO or 500 mg/kg LPC did not significantly affect Ch levels in the frontal cortex; however, 500 and 1500 mg/kg LPC combined with SCO increased its level (22.3 ± 1.1 pmol/mg tissue for LPC−, SCO+; 29.7 ± 1.2 pmol/mg tissue for LPC500, SCO+; 28.9 ± 1.1 pmol/mg tissue for LPC1500, SCO+). LPC dose dependency was not observed for ACh or Ch levels in the frontal cortex.

**Figure 3:**
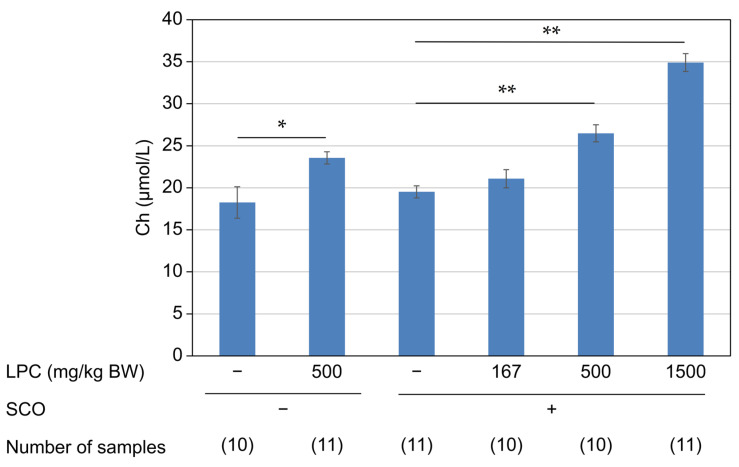
Plasma Ch levels after oral administration of LPC. Plasma Ch levels were measured 6 h after LPC administration (167, 500, and 1500 mg/kg BW, p.o.)/1.5 h after SCO injection (1 mg/kg BW, i.p.). Data are presented as mean ± SEM. *: *p* < 0.05, **: *p* < 0.01.

Although SCO treatment did not affect the plasma Ch concentration, LPC administration dose dependently increased its concentration (*p* < 0.0001; LPC−, SCO+: 19.5 ± 0.7 µmol/L; LPC167, SCO+: 21.1 ± 1.1 µmol/L; LPC500, SCO+: 26.5 ± 1.0 µmol/L; and LPC1500, SCO+: 34.9 ± 1.1 µmol/L; Figure 3). The plasma concentrations of the measured LPC species were unaffected by the SCO or the LPC treatments (Table 2).

### 3.2. Experiment 2 (LPC-Containing Food Additives and GPC)

**Figure 4:**
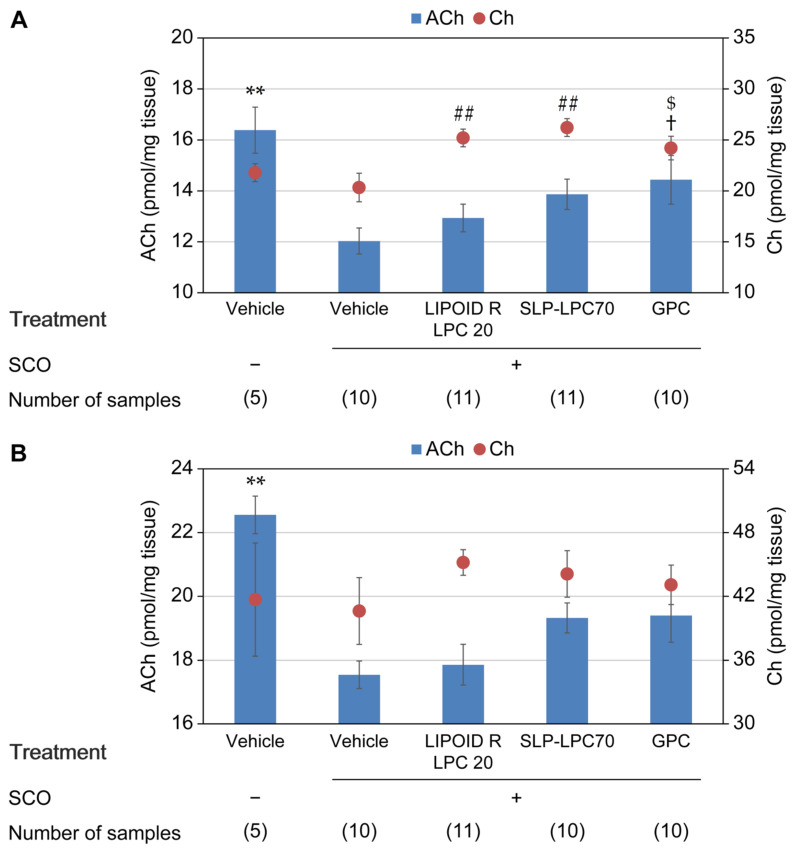

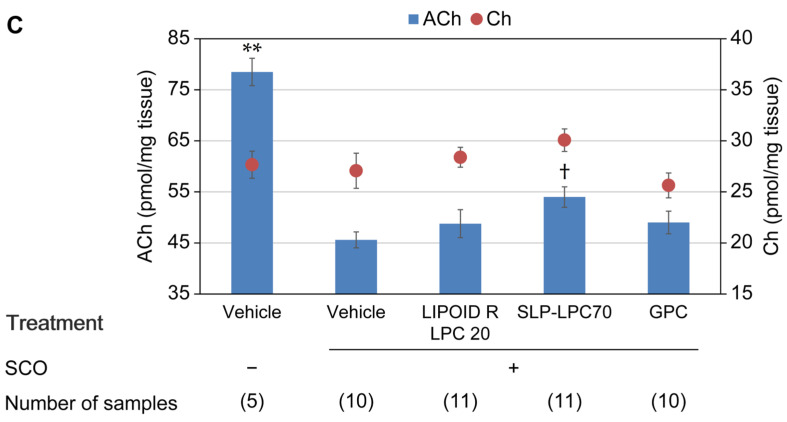
ACh and Ch levels in the brain after oral administration of LPC-containing food additives or GPC. ACh and Ch in the frontal cortex (**A**), hippocampus (**B**), and striatum (**C**) were quantified 6 h after sample administration (100 mg Ch equivalent/kg BW, p.o.)/1.5 h after SCO injection (1 mg/kg BW, i.p.). Data are presented as mean ± SEM. †: *p* < 0.1, **: *p* < 0.01 for ACh vs. Vehicle, SCO+; $: *p* < 0.1, ##: *p* < 0.01 for Ch vs. Vehicle, SCO+.

The ACh and Ch levels in the brain are shown in Figure 4. SCO-induced ACh depletion was observed in the frontal cortex, hippocampus, and striatum. In the frontal cortex, SLP-LPC70 and GPC increased ACh levels with SCO treatment; however, the differences did not attain statistical significance (12.0 ± 0.5 pmol/mg tissue for SCO-alone; 13.9 ± 0.6 pmol/mg tissue for SLP-LPC70, *p* = 0.215; and 14.4 ± 1.0 pmol/mg tissue for GPC, *p* = 0.065). LIPOID R LPC 20 and SLP-LPC70 significantly elevated Ch levels with SCO treatment (20.3 ± 1.4 pmol/mg tissue for SCO-alone; 25.2 ± 0.9 pmol/mg tissue for LIPOID R LPC 20, *p* = 0.008; and 26.2 ± 0.9 pmol/mg tissue for SLP-LPC70, *p* = 0.002). In the hippocampus, SLP-LPC70 and GPC slightly increased ACh and Ch levels; however, no significant differences were observed. In the striatum, only SLP-LPC increased ACh levels with SCO treatment (45.6 ± 1.6 pmol/mg tissue for SCO-alone and 54.0 ± 2.0 pmol/mg tissue for SLP-LPC70, *p* = 0.051); however, no significant changes in Ch levels were observed.

### 3.3. Experiment 3 (LPC-Containing Food Additives with Different Fat Contents)

Table 3 shows the levels of brain ACh and Ch and plasma Ch and LPC when SLP-LPC70 (low fat) and SLP-PasteLyso (high fat) were administered at the same LPC equivalent amount. Although no significant differences were observed between the two groups in ACh or Ch levels in the selected brain areas, the means of both ACh and Ch were higher in all brain areas when SLP-PasteLyso was administered. The plasma Ch levels were significantly lower in the SLP-PasteLyso group (*p* = 0.015). Similarly, plasma palmitoyl-LPC levels were significantly lower for the SLP-PasteLyso group (*p* = 0.003). Moreover, a significant increase in plasma linoleoyl-LPC levels was observed for the SLP-PasteLyso group (*p* = 0.022). There were no significant differences among the other LPC species and the total LPC levels.

## 4. Discussion

In our previous study, a cholinergic neuroblastoma cell line was used to demonstrate that LPC increased intracellular ACh levels [23], prompting the investigation of the cholinergic activity of LPC in vivo. To investigate the functional benefits of a wide variety of LPC species, different LPC sources, i.e., egg, soy, and rapeseed, were used for the experiments. Purified egg-derived LPC (reagent) at 500 mg/kg BW successfully mitigated SCO-induced ACh depletion in the frontal cortex of rats. A two-way ANOVA with SCO and LPC treatments as factors was additionally conducted, which showed that there was a significant SCO × LPC interaction (*p* = 0.037). However, LPC dose dependency was not observed for brain ACh levels (Figure 2). A bell-shaped dose effect relationship has been observed in experiments using GPC and other cognition-enhancing agents with different chemical structures and mechanisms of action [19,25,26]. Although the reason for the loss of response with increasing dose has not been fully elucidated, it is plausible that higher levels of the drug lead to excess ACh synthesis, triggering homeostatic mechanisms such as receptor desensitization or activation of presynaptic inhibitory receptors [19]. Moreover, an LPC-containing food additive, SLP-LPC70, tended to prevent ACh depletion when administered at 100 mg Ch equivalent/kg BW. To explore this possibility, an unpaired *t*-test analysis was conducted in addition to the main statistical analysis described in Section 2.6, which revealed that the differences in ACh values between the SCO-alone and the SCO plus SLP-LPC70 treatment groups were statistically significant for the three brain regions (*p* = 0.032, 0.012, and 0.004 for the frontal cortex, hippocampus, and striatum, respectively). GPC has been shown to restore brain ACh levels in the same experimental model [19]; however, in this study, a statistically significant difference was not observed between the SCO-alone and SCO plus GPC groups when multiplicity was considered. This discrepancy may be attributed to the differences in the applied dosage of GPC (>300 mg/kg in the previous study vs. 246 mg/kg in this study) and the sample number (up to a maximum of 29 rats in a group in the previous study). As the aim of the current study was not to prove the efficacy of GPC, we decided on the dosage based on the total amount of Ch in the test samples. When an unpaired *t*-test analysis was conducted on the differences between the two groups, statistical significance was attained for the frontal cortex (*p* = 0.040).

To explore the mechanisms by which LPC prevented ACh depletion, plasma Ch and LPC concentrations were measured in Experiments 1 and 3. The concentration of blood phospholipids could not be determined because microwave irradiation causes hemolysis. The fatty acid composition of (lyso)phospholipids in animals and plants differs depending on their origin [27,28]. Therefore, measuring palmitoyl-, stearoyl-, oleoyl-, linoleoyl-, and linolenoyl-LPCs, the predominant LPC forms in the tested reagent and food additives (Table 1), was prioritized. In Experiment 1, although LPC was applied at 1500 mg/kg BW, plasma LPC concentrations were unaffected, regardless of the fatty acid species (Table 2). However, when SLP-PasteLyso was administered in Experiment 3, the plasma concentrations of some LPC species changed significantly (Table 3). Interestingly, the LPC species whose increase was observed was linoleoyl-LPC, which was the most abundant LPC form in the soy-derived enzyme-modified lecithin used for the experiments (Table 1). Because there were no significant differences in PC and free Ch contents between the two test samples, we attributed the increased linoleoyl-LPC level to the relatively high fat content of SLP-PasteLyso (acetone-soluble matter: 55% for SLP-PasteLyso vs. 6% for SLP-LPC70). Fat has been shown to help LPC to be reacylated into PC in enterocytes and incorporated into chylomicrons [29]. As a result of increased incorporation into chylomicron PC, the proportion of LPC that is degraded into free Ch and absorbed through the portal vein decreases [21]. PC incorporated into circulation can be metabolized to LPC by lecithin-cholesterol acyltransferase (LCAT) and lipoprotein-associated phospholipase A2 (Lp-PLA2). Another possible metabolic rate is that orally ingested LPC can be absorbed intact, at least to some extent [29]. These metabolic and absorption pathways support our finding that an increase in plasma linoleoyl-LPC levels was accompanied by a decrease in plasma Ch levels. LPC species with unsaturated fatty acids in circulation are transported to the brain via the major facilitator superfamily domain-containing protein 2 (Mfsd2a) [22,30]. This supports the possibility that more linoleoyl-LPC in the blood was transferred to the brain after SLP-PasteLyso administration, resulting in higher ACh and Ch levels in the brain than SLP-LPC70. Given that Murota et al. reported that DHA-LPC was more efficiently incorporated into chylomicrons than LPC with other fatty acids [31], a direct comparison of the bioavailability between linoleoyl-LPC and DHA-LPC should be performed in future experiments.

Importantly, when blood liloleoyl-LPC levels were elevated, palmitoyl-LPC levels declined significantly. LPC acyltransferase (LPCAT), which is expressed in several organs (such as the liver and brain) and plays a role in LPC clearance [14], is likely to be involved in this phenomenon. Additionally, although an increase in total LPC concentration in the blood is associated with inflammatory/pathogenic changes [14], total LPC levels remained unchanged even when linoleoyl-LPC levels increased and when high doses of LPC (up to 1500 mg/kg BW) were administered. These findings are noteworthy from a safety perspective. When we decided on LPC doses for this study, we referred to a 13-week toxicity study of enzyme-modified lecithin containing up to 30% LPC. In the study, the no-observed-adverse-effect level (NOAEL) was estimated to be 20.5 g/kg [32]. Therefore, we consider that the LPC doses applied in the current study as safe to consume and unlikely to cause systemic inflammation. Future examinations of the brain histology and the blood LPA concentration under the condition that LPC functions as a cholinergic agent would further dispel the safety concerns pertaining to LPC consumption.

In association with the benefit of Ch intake in the form of LPC, a previous study showed that DHA-LPC more efficiently increased the blood DHA-LPC concentration than DHA-PC given at the same DHA amount, and the increase in blood DHA-LPC was accompanied by the enrichment of brain-derived neurotrophic factor (BDNF) in the brain. This is because DHA-PC is hydrolyzed during the digestion process and releases free DHA, which is used for triglyceride production instead of being incorporated into PC. Dietary DHA-LPC, on the other hand, tends to be more absorbed either in the PC or LPC form [7]. The bioavailability of the Ch moiety in the brain was not shown in the study, but another report showed that the ingested LPC in the eicosapentaenoic acid-bound form served as a Ch source for the brain [33]. Given that the PC content of SLP-LPC70 was only 2%, whereas that of LIPOID R LPC 20 was 26%, the relatively lower activity of LIPOID R LPC 20 in increasing the brain ACh may be attributable to the relatively higher PC content (Figure 4).

Even when Ch-containing compounds are absorbed as free Ch, they are transported to the brain, as well as LPC bound to unsaturated fatty acid, thereby increasing Ch availability in the brain. However, the brain Ch concentration is not necessarily correlated with the brain ACh concentration (reviewed in [34]). This notion was supported by our results. Moreover, it has been shown that PC is complementarily used for ACh biosynthesis, and brain PC availability is important for ACh production [35]. Notably, LPC in the brain can be converted to PC for membrane synthesis by LPCAT [36]. Furthermore, it is intriguing to know that the meta-analyses on the patients with Alzheimer’s disease showed significantly lower levels of Ch-containing lipids in the brain and PC in blood [37]. Considering that LPC treatment elevated ACh levels in cholinergic neurons [23], brain LPC availability may also be related to ACh synthesis in addition to brain Ch and PC, which suggests the additional benefit of LPC supplementation. It is clear that brain LPC levels after LPC administration should be quantified to investigate this possibility.

The possible advantage of LPC consumption over GPC is discussed. Some studies have demonstrated that PC consumption results in a lower increase in blood trimethylamine *N*-oxide (TMAO) concentration than Ch and GPC consumption at the same Ch-equivalent dose [38,39]. TMAO is produced by bacterial degradation prior to the absorption of Ch and is associated with atherosclerosis [40]. The same phenomenon may be observed for LPC, because part of the ingested LPC follows the metabolic pathway of PC [21]. However, we could not investigate this possibility, as we prioritized evaluating ACh concentrations in the hippocampus and striatum and did not collect blood samples during Experiment 2.

This study had some limitations. First, brain and blood concentrations were evaluated only at a single time point. The sampling timing was determined based on previous studies. Essentially, plasma and brain Ch levels peaked 6 h after PC consumption [41,42]. Moreover, while the maximal incorporation of GPC-derived Ch into the brain is approximately 8–10 h after oral administration of GPC, the brain ACh recovery under SCO treatment was observed even 5 h after administration [19,43]. As the LPC-containing food additives administered at the same Ch or LPC equivalent doses differed in their contents, and the same volume of test solutions were administered to rats, differences in their osmotic pressures could affect the speed of gastric emptying [44], absorption pathway (portal vein or lymphatic vessel) [45,46], and metabolic rate of LPC. Therefore, peaks in the brain ACh concentrations for some test samples may not have been detected. The difference in osmotic pressure may also explain the lower activity of LIPOID R LPC 20. The total Ch content (including free and [lyso]phospholipid-bound forms) of LIPOID R LPC 20 was estimated to be 5%, while that of SLP-LPC70 was estimated to be 8%. Therefore, the administered dose of LIPOID R LPC 20 was 1.6 times that of SLP-LPC70. Second, only brain ACh and Ch concentrations were measured to assess the cholinergic activity of LPC. Behavioral tests to evaluate cognitive and psychological functions should be conducted as the next step to determine the possible benefits of preventing ACh depletion.

To the best of our knowledge, this is the first in vivo study to demonstrate that orally administered LPC species other than DHA-LPC function as cholinergic agents, and their activity is comparable to that of GPC. Given that GPC has been demonstrated to enhance human cognitive function [3,4], LPC should be further investigated as a candidate for new functional foods and drugs using different experimental models.

## Figures and Tables

**Table 1:** Fatty acid compositions of the LPC contained in the reagent and food additives used in this study.

	LPC Species (%)				
	Palmitoyl	Stearoyl	Oleoyl	Linoleoyl	Linolenoyl
LPC reagent (egg-derived)	71.9	21.5	6.6	ND	ND
LIPOID R LPC 20 (rapeseed-derived)	16.6	ND	53.5	24.9	5.0
SLP-LPC70 (soy-derived)	25.3	5.2	9.6	53.1	6.8
SLP-PasteLyso (soy-derived)	29.0	5.9	9.8	46.6	8.8

Palmitoyl-, stearoyl-, oleoyl-, linoleoyl-, and linolenoyl-LPCs were quantified, and each ratio to the total LPC is presented. Quantification was performed thrice using the same lot, and the average value was used. ND, not detected.

**Table 2:** Plasma LPC concentrations after oral administration of egg-derived LPC.

LPC Species	Treatment					
	LPC− SCO−	LPC500 SCO−	LPC− SCO+	LPC167 SCO+	LPC500 SCO+	LPC1500 SCO+
Palmitoyl (µmol/L)	123.3 ± 5.5	113.0 ± 3.9	120.8 ± 4.0	119.7 ± 4.5	118.0 ± 5.7	116.0 ± 3.4
Stearoyl (µmol/L)	70.1 ± 4.9	62.5 ± 3.6	64.7 ± 2.3	66.7 ± 3.1	64.9 ± 3.5	62.3 ± 2.1
Oleoyl (µmol/L)	21.8 ± 0.9	18.3 ± 0.8	19.2 ± 0.9	19.0 ± 1.1	19.0 ± 1.0	17.6 ± 0.8
Linoleoyl (µmol/L)	99.7 ± 4.3	89.0 ± 4.6	93.6 ± 4.5	92.6 ± 4.2	86.9 ± 3.6	83.4 ± 4.3
Total (µmol/L)	314.8 ± 14.6	282.8 ± 12.5	298.4 ± 10.5	298.0 ± 12.1	288.8 ± 13.3	279.2 ± 9.6

Palmitoyl-, stearoyl-, oleoyl-, and linoleoyl-LPCs in plasma were quantified 6 h after LPC administration (167, 500, and 1500 mg/kg BW, p.o.)/1.5 h after SCO injection (1 mg/kg BW, i.p.). Total LPC was calculated as the sum of the four LPC species. Linolenoyl-LPC was below the determination limit. Data are presented as the mean ± SEM (n = 10, 11).

**Table 3:** Brain ACh and Ch and plasma LPC concentrations after oral administration of soy-derived LPC-containing food additives with varying fat content.

		Treatment		
		SLP-LPC70 SCO+	SLP-PasteLyso SCO+	
Frontal cortex	ACh (pmol/mg tissue)	13.6 ± 0.4	14.1 ± 0.5	
	Ch (pmol/mg tissue)	24.1 ± 1.4	25.9 ± 1.2	
Hippocampus	ACh (pmol/mg tissue)	17.6 ± 0.3	18.4 ± 0.5	
	Ch (pmol/mg tissue)	47.2 ± 2.7	48.2 ± 0.9	
Striatum	ACh (pmol/mg tissue)	51.0 ± 1.1	55.3 ± 3.3	
	Ch (pmol/mg tissue)	29.2 ± 1.3	29.6 ± 1.3	
Plasma	Ch (µmol/L)	24.9 ± 0.6	22.1 ± 0.8	*
	Palmitoyl-LPC (µmol/L)	120.5 ± 2.7	104.8 ± 3.8	**
	Stearoyl-LPC (µmol/L)	65.5 ± 2.5	60.3 ± 2.3	
	Oleoyl-LPC (µmol/L)	18.7 ± 1.1	17.4 ± 1.0	
	Linoleoyl-LPC (µmol/L)	94.6 ± 3.8	110.3 ± 5.1	*
	Total LPC (µmol/L)	299.3 ± 9.0	292.9 ± 11.2	

ACh and Ch in the frontal cortex, hippocampus, and striatum as well as palmitoyl-, stearoyl-, oleoyl-, and linoleoyl-LPCs in plasma were quantified 6 h after sample administration (500 mg LPC equivalent/kg BW, p.o.)/1.5 h after SCO injection (1 mg/kg BW, i.p.). Total LPC was calculated as the sum of the four LPC species. Linolenoyl-LPC was below the determination limit. Data are presented as the mean ± SEM (n = 9–11). *: *p* < 0.05, **: *p* < 0.01.

## Data Availability

The data presented in this study are available in Datasets S1–S3.

## Supplementary Materials

The following supporting information can be downloaded at: https://www.mdpi.com/article/10.3390/nu15163618/s1, Dataset S1: Experiment 1, brain tissue analysis; Dataset S2: Experiment 1, plasma analysis; Dataset S3: Experiments 2 and 3.
